# Systems to identify potentially inappropriate prescribing in people with advanced dementia: a systematic review

**DOI:** 10.1186/s12877-016-0289-z

**Published:** 2016-05-31

**Authors:** Domenica Disalvo, Tim Luckett, Meera Agar, Alexandra Bennett, Patricia Mary Davidson

**Affiliations:** Centre for Cardiovascular and Chronic Care, Faculty of Health, University of Technology Sydney (UTS), 235-253 Jones St, Ultimo, NSW 2007 Australia; Ingham Institute of Applied Medical Research, Sydney, NSW Australia; HammondCare, Sydney, NSW Australia; South Western Sydney Clinical School, University of New South Wales (UNSW), Sydney, NSW Australia; NSW Therapeutic Advisory Group, Sydney, NSW Australia; St Vincent’s Hospital, Sydney, Australia; School of Nursing, Johns Hopkins University (JHU), Baltimore, USA

**Keywords:** Dementia, Polypharmacy, Deprescribing, Inappropriate prescribing, Medication review, Palliative care

## Abstract

**Background:**

Systems for identifying potentially inappropriate medications in older adults are not immediately transferrable to advanced dementia, where the management goal is palliation. The aim of the systematic review was to identify and synthesise published systems and make recommendations for identifying potentially inappropriate prescribing in advanced dementia.

**Methods:**

Studies were included if published in a peer-reviewed English language journal and concerned with identifying the appropriateness or otherwise of medications in advanced dementia or dementia and palliative care. The quality of each study was rated using the STrengthening the Reporting of OBservational studies in Epidemiology (STROBE) checklist. Synthesis was narrative due to heterogeneity among designs and measures. Medline (OVID), CINAHL, the Cochrane Database of Systematic Reviews (2005 – August 2014) and AMED were searched in October 2014. Reference lists of relevant reviews and included articles were searched manually.

**Results:**

Eight studies were included, all of which were scored a high quality using the STROBE checklist. Five studies used the same system developed by the *Palliative Excellence in Alzheimer Care Efforts (PEACE)* Program. One study used number of medications as an index, and two studies surveyed health professionals’ opinions on appropriateness of specific medications in different clinical scenarios.

**Conclusions:**

Future research is needed to develop and validate systems with clinical utility for improving safety and quality of prescribing in advanced dementia. Systems should account for individual clinical context and distinguish between deprescribing and initiation of medications.

## Background

Advanced dementia infers a range of physical and psychosocial needs [1]. A palliative approach that maximises comfort is considered best practice [2]. Medication use should be focused on symptom relief and quality of life rather than treating secondary conditions where burden is likely to outweigh clinical benefit [2].

Most research on potentially inappropriate prescribing has focused on the elderly rather than dementia specifically. The harm/benefit risk ratios of numerous medications are unfavourably affected by age-related changes in pharmacokinetic and pharmacodynamic parameters [3]. Biological changes can result in medications having longer durations of action, greater risks of toxicity, and increased frequencies of adverse effects.

Several systems for identifying potentially inappropriate medications in older adults have been developed to operationally define the harm/benefit risk in clinical practice and research [4, 5]. These systems have been applied in early but not advanced dementia [6, 7]. Generalizability to people with advanced dementia is limited by pathophysiological changes as dementia progresses and the fact that systems have not been developed for use where goals of care are palliative. In advanced dementia, there is an exaggerated decrease in total body water and muscle and an increase in relative adipose tissue [8]. These changes are additional to the changes due to aging and have a direct and variable impact on the metabolism of drugs [9]. This means that individuals with advanced dementia may be more prone to adverse drug effects and drug-drug interactions than other older people [10]. People with advanced dementia are also less able than others to report adverse effects or to be involved in decision-making about whether to initiate or withdraw medications. Finally, individuals with advanced dementia have typically been excluded from research examining quality use of medications in older populations, limiting evidence regarding benefits and harms. Identifying potentially inappropriate medications to guide prescribing practice for people with advanced dementia is therefore likely to face challenges over and above those for older populations more generally.

A review by Parsons et al. (2010) summarised literature on specific medication types proposed to be potentially inappropriate for people with dementia nearing the end of life, and examined decision-making regarding medication discontinuation [9]. Potentially inappropriate medications were identified to include anticholinesterase inhibitors, memantine, antipsychotics, statins, antibacterials, antihypertensives, antihyperglycaemic agents, anticoagulants and medications to manage osteoporosis. Parsons et al. highlighted the lack of guidance on identifying potentially inappropriate medications and when and how to safely discontinue medications at the end of life.

A distinct but related concept is polypharmacy. Polypharmacy refers to the combination of multiple medications which may lead to cumulative adverse effects and antagonistic drug-drug interactions where a worse adverse effect is produced than either drug could have caused alone [11]. Polypharmacy can lead to worse side effects in the same domain (e.g. if receiving several psychoactive medications) or more side effects across different domains (e.g. if receiving a psychoactive medication and a blood pressure medication). Each of the medications involved may or may not be deemed potentially inappropriate on their own.

The current authors set out to update the review by Parsons et al. using a more rigorous systematic methodology and specifically aiming to identify and synthesise any published systems and recommendations for identifying potentially inappropriate prescribing in people with advanced dementia.

## Methods

This systematic review was undertaken in adherence with the Preferred Reporting Items for Systematic Reviews and Meta-analyses (PRISMA) Statement [12].

### Eligibility criteria

Articles needed to be published in a peer-reviewed English language journal and report on a system or recommendations for identifying the appropriateness or otherwise of medications in advanced dementia or dementia and palliative care.

### Information sources

Electronic databases Medline (OVID), CINAHL, the Cochrane Database of Systematic Reviews (2005 – August 2014) and AMED were searched in October 2014. Reference lists of the review by Parsons et al. (2010) and included articles were searched by hand.

### Search

Database searches used keyword searches and medical subject headings (MeSH) based on terms used by Parsons et al. (2010) but further terms were also added as detailed in Table 1.

**Table 1:** Electronic database search terms used to find articles reporting on systems to identify potentially inappropriate prescribing in people with advanced dementia

Parsons et al. (2010) [9] search terms:	
medication(s) medicine(s)’ discontinue, discontinuation withhold(ing) withheld withdraw	withdraw(al) dementia severe dementia end of life palliative care nursing home
Terms recommended by the Australian online palliative care knowledge network, CareSearch [30] were further included:	
Inappropriate prescri* inappropriate med* medication management medication review medic* of risk terminal care	hospices hospice patients hospice care deprescrib*^α^ prescribing patterns polypharmacy

^α^The term “deprescribing” has been coined to describe the process of tapering or withdrawing drugs with the goal of managing polypharmacy and improving outcomes [31], *Truncation used to ensure all variations and different spelling of words were retrieved

### Study selection

Two researchers (DD, TL) independently applied the eligibility criteria to 10 % of search results and checked inter-rater reliability. After finding 100 % agreement, a single investigator (DD) rated the remaining 90 % articles alone. Full-texts were reviewed where a decision could not be made on abstract and title alone.

### Data collection and items

Data were extracted from eligible studies by a single researcher (DD) using a standardised template. Data items extracted included: study design, aims, setting, sample size and characteristics, details of the approach taken to identifying inappropriate medications, and outcome variables related to inappropriate prescribing.

### Risk of bias in individual studies

The quality of each study was rated independently by two researchers (DD and TL) using criteria from the STrengthening the Reporting of OBservational studies in Epidemiology (STROBE) [13]. Any disagreements were resolved via discussion.

### Synthesis

Expected heterogeneity among designs and methods meant that synthesis needed to be narrative rather than via meta-analysis. Methods for narrative synthesis were based on techniques described by Popay et al. (2006) [14].

## Results

### Study selection

**Fig. 1:**
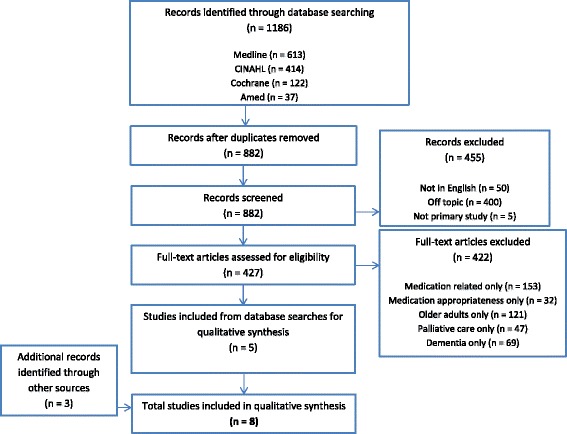
Flowchart depicting inclusion/exclusion

Database searches identified 882 records once duplicates were removed. Five articles were included for analysis from electronic database searches [15–19], and a further three articles were additionally identified through hand searching [20–22]. See Fig. 1 for more details.

#### Study characteristics

Characteristics of the eight studies included in this review are summarised in Table 2. The studies variously aimed to: 1) determine the prevalence of potentially inappropriate prescribing in aged care residents with advanced dementia [15–18, 20, 22], 2) identify the factors associated with likelihood of potentially inappropriate medications [16–18, 20, 22], and 3) explore the perceptions of healthcare professionals regarding factors determining medication-related decision-making in this population [19, 21].

**Table 2:** Summary of eight studies included in the review which use a system to identify potentially inappropriate prescribing in advanced dementia or dementia in palliative care

First Author, Year	Country	Aim(s)	Design	N at baseline	Setting	Approach to identify inappropriate medications	Medication variables	Results
Studies which use number of medications as indication of potentially inappropriate prescribing i.e. polypharmacy								
Blass et al. 2008 [15]	USA (Baltimore)	Identify how medication usage changed over time as resident with advanced dementia moves toward death, and identify correlates of increased medication usage.	Prospective cohort study (longitudinal)	125 residents	3 nursing homes	Number of medications prescribed i.e. polypharmacy.	Number of medications prescribed (regular + prn) at baseline, and factors associated with total number of medications.	Residents prescribed 14.6 medications each. Increase in palliative medicines i.e. opiates and a decrease in antibiotics, anti-dementia agents, cardiovascular agents and psychotropics as death approaches. No change in the number of medications given over time.
Studies using explicit criteria to identify potentially inappropriate prescribing								
Holmes et al. 2008 [16]	USA	Evaluate the feasibility of developing consensus recommendations for appropriate prescribing for patients with advanced dementia.	Modified Delphi consensus panel (and medication record audit) (cross-sectional)	34 patients	3 long term care facilities	Using modified Delphi process (12 geriatricians), medications categorised for use in palliative care patients with advanced dementia; never, rarely, sometimes and always appropriate.	Determine frequency of inappropriate medication prescribing, using in-house developed explicit criteria.	Patients taking 6.5 medications each. Six patients taking ten or more medications daily. 29 % of patients taking a medication considered never appropriate.
Tjia et al. 2010 [17]	USA (Chicago)	Describe the pattern and factors associated with daily medication use in nursing home residents with advanced dementia.	Prospective cohort study (longitudinal)	323 residents	22 nursing homes	Drugs of questionable benefit i.e. ‘never appropriate’ according to medications classified by Holmes et al. 2008.	Resident characteristics associated with the use of daily medications and drugs deemed inappropriate.	Male, shorter length of stay at facility (<1 year), higher functional ability and diabetes independently associated with inappropriate drug use. Having a DNR order independently associated with a lower likelihood of inappropriate drug use.
Colloca et al. 2012 [20]	7 EU countries (Czech Republic, England, Finland, France, Germany, Italy, The Netherlands) and Israel	Identify prevalence and factors associated with use of inappropriate drugs in older adult patients with severe cognitive impairment.	Medication chart audit (cross-sectional)	1449 residents	57 nursing homes	The use of drugs classified as rarely or never appropriate by criteria developed by Holmes et al. 2008.	Inappropriate drug use defined as rarely or never appropriate in patients with severe cognitive impairment based on the Holmes criteria published in 2008.	Inappropriate drug use in 643 (44.9 %) of residents. Most commonly prescribed inappropriate drugs were lipid-lowering agents (9.9 %), antiplatelet agents (9.9 %), Ach inhibitors (7.2 %) and antispasmodics (6.9 %). Inappropriate drug use associated with diabetes, HF, stroke, recent hospitalization. An inverse relationship between inappropriate drug use and geriatrician at facility.
Toscani et al. 2013 [18]	Italy	Assess and compare treatments and prescriptions of patients with advanced dementia cared for in nursing homes and in home care and assess their appropriateness from a palliative care perspective.	Baseline data from multicentre prospective observational cohort study	245 residents	Nursing homes	Used criteria developed by Holmes et al. 2008.	The appropriateness of each prescription assessed according to the Holmes et al. 2008 classification.	Patients received 4.1 medications on average (range 0–13). Laxatives, antipsychotics, and anxiolytics were the most frequently prescribed in the nursing homes. 8.1 % of residents receiving at least one analgesic.
Tjia et al. 2014 [22]	USA	Estimate the prevalence of medications with questionable benefit used by nursing home residents with advanced dementia.	Medication record audit (cross-sectional)	5406 residents	Nursing homes	Medications deemed never appropriate for use in advanced dementia according to criteria developed by Holmes et al. 2008.	Use of medication of questionable benefit in advanced dementia based on previously published criteria and mean 90-day expenditures due to these medications per resident.	53.9 % of residents receiving at least one medication with questionable benefit. Anticholinesterase inhibitors (36.4 %), memantine (25.2 %) and lipid-lowering agents (22.4 %) most commonly prescribed medications with questionable benefit.
Other approaches to identify inappropriate prescribing								
Shega et al. 2009 [19]	USA	Describe hospice medical directors practice patterns and experiences in the use and discontinuation of anticholinesterase inhibitors and memantine in hospice patients with dementia.	Mail survey (cross-sectional)	152 hospital medical directors	Hospice care	N/A	Associations between the likelihood of survey response and participant characteristics. Comparisons analysing whether or not a physician would recommend medication discontinuation based upon reported clinical benefit of anticholinesterase inhibitors and memantine use.	Of the respondents, 75 % and 33 % reported that at least 20 % of patients were taking anticholinesterase inhibitor or memantine at hospice admission. 80 % of respondents would recommend discontinuation of these agents, however, a subset believe they stabilize cognition (22 %), decrease challenging behaviours (28 %), maintain patient function (22 %,) reduce caregiver burden (20 %) and improve caregiver quality of life (20 %).
Parsons et al. 2014 [21]	NI (Northern Ireland), RoI (Republic of Ireland)	Evaluate the extent to which patient-related factors and physicians’ country of practice influenced decision making regarding medication use in patients with end-stage dementia.	Factorial survey design	662 health professionals	Community, nursing home, hospital	Medications selected due to contradictory evidence available to guide practice or because they have been identified in the limited literature as potentially inappropriate for individuals with advanced dementia: antibiotics, anticholinesterase inhibitors, memantine, lipid-lowering agents and antipsychotics.	Assess physician decision making regarding withholding or continuation/discontinuation of key medications in patients with end-stage dementia.	Considerable variability found regarding initiating/withholding antibiotics and continuing/discontinuing anticholinesterase inhibitors and memantine hydrochloride. Less variability found in decision making regarding lipid-lowering agents and antipsychotics. Patient place of residence and physician country of practice had the strongest and most consistent effects on decision making.

Five studies were undertaken in the USA [15–17, 19, 22] and three were undertaken in European countries [18, 20, 21].

Study designs included two cross-sectional surveys [19, 21], three prospective cohort studies [15, 17, 18], one of which reported the cross-sectional results of medication data collected at baseline [18], two retrospective clinical record audits [20, 22], and one combining a retrospective clinical record audit with a consensus panel component [16].

Six studies analysed medication data from a total of 7457 participants with advanced dementia, their age ranging from 57 to 100 years of age and the majority being female, ranging from 55.2 % [15] to 87.5 % [17] of their samples. Of these, four studies focused solely on nursing homes [15–17, 20, 22] while two also included people with advanced dementia receiving home care [18, 20].

### Risk of bias within studies

The eight studies included in the systematic review were generally of high quality as rated by STROBE criteria, complying with 76 % [16] to 100 % [17, 22] of criteria. However, Toscani et al. (2013) did not indicate the study’s design in the title or abstract [18], and Colloca et al. (2012) did not sufficiently explain a larger study’s design from which their data were drawn [20]. Four studies did not give a rationale for sample size [15, 16, 20, 21]. Three studies did not attempt to address potential sources of bias [15, 16, 21]. These same three studies also provided limited descriptions of statistical methods or how they dealt with missing data. Three studies did not provide unadjusted results for their multivariate analyses [16, 18, 20], and one controlled only for gender and age rather than other socio-demographic, clinical and nursing home variables [18]. Two studies did not discuss the generalizability of their results [18, 19].

### Synthesis of results

Five of the eight studies [16–18, 20, 22] used the same system for identifying potentially inappropriate medications – that was developed by the *Palliative Excellence in Alzheimer Care Efforts (PEACE)* Program reported by Holmes et al. (2008) [16]. In the PEACE program, medications were audited for 34 patients with advanced dementia where a palliative approach was deemed appropriate. In a three-round modified Delphi process, 12 geriatricians rated each medication identified via the audit as ‘never’, ‘rarely’, ‘sometimes’ or ‘always’ appropriate. Consensus for a medication or medication class was defined as agreement on categorisation by >50 % (i.e. at least 7/12) participants. See Table 3 for drug classes in each category according to the final consensus.

**Table 3:** Appropriateness of medications as defined by PEACE consensus panel

Always appropriate		
Antidiarrheals	Antiepileptic drugs	Expectorants
Laxatives	Anxiolytics	Lubricating eye drops
Antiemetics	Narcotic analgesics	Pressure ulcer products
Inhaled bronchodilators	Nonnarcotic analgesics	Lidoderm
Sometimes appropriate		
Proton pump inhibitors	Antidepressants	Insulin
Histamine-2 receptor blockers	Tricyclic antidepressants	Antihistamines
Beta-blockers	Antibacterials	Decongestants
Calcium channel blockers	Antivirals	Electrolytes
Diuretics	Antiparasitic agents	Nutritional supplements
Angiotensin-converting enzyme inhibitors and angiotensin receptor blockers	Antifungal creams	Antiglaucoma drops
Nitroglycerin	Oral hypoglycaemics	Anti-inflammatory eye drops
Mucolytics	Thyroid hormones	Capsaicin
Inhaled corticosteroids	Antithyroid medications	Allopurinol
Antipsychotics	Corticosteroids	Colchicine
Rarely appropriate		
Alpha blockers	Antiandrogens	Appetite stimulants
Digoxin	Bisphosphonates	Bladder relaxants
Clonidine	Mineralocorticoids	Tamsulosin
Antiarrhythmics	Heparin and low molecular-weight heparins	Antispasmodics
Hydralazine	Warfarin	
Never appropriate		
Lipid-lowering medications	Memantine	Cytotoxic chemotherapy
Antiplatelet agents, excluding aspirin	Antiestrogens	Hormone antagonists
Leukotriene receptor antagonists	Sex hormones	Immunomodulators
Acetylcholinesterase inhibitors		
No consensus		
Aspirin	Meclizine	Bladder stimulants
Sedatives and hypnotics	Vitamins	Iron
Central nervous system stimulants	Mineral supplements	Finasteride
Muscle relaxants	Calcitonin	Red blood cell colony stimulating factors

Sourced from Holmes et al. (2008) [16]

Following Holmes and colleagues’ preliminary study [16], four other international studies utilised [17, 18, 20, 22] the PEACE criteria to rate the appropriateness of medications taken by large cohorts of aged care residents with advanced dementia and examine predictors of taking ‘never’ appropriate medications among socio-demographic and clinical variables. See Table 4 for a summary of these studies’ samples and results.

**Table 4:** Results from studies utilising PEACE criteria to determine appropriateness of medications in individuals with advanced dementia

Authors	Country	N at baseline	Mean (SD) medications per resident at baseline	N (%) using ‘never’ appropriate medications^a^	Most common ‘never’ appropriate medications	Factors associated with using ‘never’ appropriate medications	Factors measured but did not show an association with using ‘never’ appropriate medications
Holmes et al. 2008 [16]	USA	34	6.5 (2.7)	10 (29 %)	Cardiovascular agents	*Not measured*	*Not measured*
Tjia et al. 2010 [17]	USA	323	6.2 (3.33)	121 (37.5 %)	Lipid lowering agents Anticholinesterase inhibitors	Male Shorter length of stay^#^ Higher functional ability^c^ Diabetes mellitus DNH order (inverse)	Age Ethnicity (non-white race vs white) In special care dementia unit Dementia due to Alzheimer’s disease Test for Severe Impairment score > 0^d^ Cardiovascular disease^e^ Cancer Acute illness in prior 90 days^f^ Recent hospitalization^g^ Recent physician/nurse professional visits in prior 90 days No feeding tube No hospice referral
Colloca et al. 2012 [20]	7 EU countries (Czech Republic, England, Finland, France, Germany, Italy, The Netherlands) and Israel	1449	4 *(not reported)*	388 (26.8 %)	Lipid lowering agents Antiplatelets Anticholinesterase inhibitors	Stroke	Age Gender Shorter length of stay^b^ Ethnicity (non-white vs white) ADL Hierarchy Scale score^h^ Behavioural symptoms Falls Number of diseases Ischaemic heart disease Diabetes Heart failure Cancer Parkinson’s disease Urinary tract infections Pneumonia Fractures Recent hospitalization^g^ Presence of a geriatrician Presence of a pharmacist
Toscani et al. 2013 [18]	Italy	245	*Not reported*	9 (2.2 %)	Antihypertensives Antiplatelets	*Not measured*	*Not measured*
Tjia et al. 2014 [22]	USA	5406	7.33 (3.5)	2911 (53.9 %)	Lipid lowering agents Memantine Anticholinesterase inhibitors	High facility use of feeding tubes	Age Gender Ethnicity (non-white vs white) DNR order Hospice enrolment Whether Medicaid is primary payor In special care dementia unit Recent hospitalization^g^ Recent physician visit (last 14 days) Diabetes mellitus Heart Failure Hypertension Stroke Osteoporosis Depression Nutritional problems Oral problems Behavioural issues Functional status

^a^as defined by the *Palliative Excellence in Alzheimer Care Efforts (PEACE)* criteria reported by Holmes et al. (2008) [13]

^b^Less than 1 year in nursing home

^c^Bedford Alzheimer Nursing Scale – Severity Subscale, possible range 7–28, higher scores indicate greater functional disability

^d^possible range 0–24, lower scores indicate greater cognitive impairment

^e^Cardiovascular disease includes history of coronary artery disease and cerebrovascular accident

^f^Acute illnesses include infectious episodes myocardial infarction, stroke, any bone fracture, gastrointestinal bleed, and seizure

^g^any hospitalization occurring in the last 90 days

^h^ADL hierarchical scale score ranges from 0 (no impairment) to 6 (total dependence in self-care)

*ADL* Activities of Daily Living, *DNH* Do Not Hospitalize, *DNR* Do Not Resuscitate, *N* number, *SD* standard deviation

Blass et al. (2008) used a more rudimentary index of potentially inappropriate prescribing in people with advanced dementia based purely on number of medications [15]. The study identified that nursing home residents with advanced dementia received a mean of 14.6 medications (±7.4) and that, as residents approached death, the type but not number of medications altered. The study identified an increase in medications for symptom control (i.e. opioids and laxatives) and a decrease in medications for comorbid conditions (i.e. antibiotics, anti-dementia drugs, cardiovascular agents and psychotropic agents).

Two studies by Shega et al. (2009) and Parsons et al. (2014) explored factors influencing medication-related decisions by physicians (hospital medical directors [19], general practitioners and hospital physians [21]), specifically their continuation or discontinuation in dying patients with dementia [19, 21]. Physicians from both studies recommended discontinuation of anticholinesterase inhibitors and memantine because of perceived lack of clinical benefit during end-stage of illness [21], but were less likely to recommend this if there was any indication that they stabilised cognition, reduced challenging behaviours or maintained patient function [19]. Physicians also recommended discontinuing quetiapine and simvastatin because of a perceived lack of indication and/or risk of adverse effects such as confusion [21]. Emphasis was placed on ensuring patient comfort and symptom management and reducing polypharmacy and preventative treatments.

## Discussion

This systematic review identified only one system for identifying potentially inappropriate medications in people with advanced dementia that had any degree of validation – the PEACE criteria developed by Holmes et al. (2008) [16]. A second system we identified relied on number of medications alone [15]. Finally, two other studies have sought to understand the decision-making process of health professionals when determining the appropriateness of medications in end-stage dementia.

Whilst providing a useful foundation, the PEACE criteria are limited in a number of ways. Holmes et al. (2008) themselves identified a need for further validation of the system by means of a larger sample of medication data and a more representative expert panel of health professionals. Their expert informants were all geriatricians from the University of Chicago. Moreover, Holmes et al. (2008) did not report informants’ rationale for medication classification within the system. Authors using the PEACE criteria since have highlighted its ‘one size fits all’ approach and the importance of taking into account each older individual’s life expectancy [17, 18, 20], comorbidities, symptom experience [16, 22] and goals of care [18]. These concerns are especially applicable to the PEACE categories of ‘sometimes’ and ‘rarely’ appropriate, which are of limited usefulness without a better understanding of factors influencing decision-making.

Studies using the PEACE criteria suggest insights into how this system might be refined and validated in the future. Percentages of residents ‘never’ appropriate medications varied between studies. In addition to differences in prescribing cultures between countries and organisations included in these studies, differences in rates of never appropriate medications may have resulted in part from variability in the methods used to define advanced dementia and code medications. Three studies used the Cognitive Performance Scale (CPS) [17, 20, 22] to define advanced dementia while two others used the Functional Assessment Staging Tool (FAST) [16, 18]. With regard to coding medications, two studies used the Anatomical Therapeutic Classification (ATC) System [18, 20] and two used the British National Formulary [17, 22]; both these approaches differed from the original study, which utilised the British National Formulary, United States Pharmacopeia and National Formulary and the Lexi-Comp alphabetical drug index [16]. While Colloca et al. did not provide a list of ATC codes they included, Toscani et al. indicated that ATC codes (beginning with N06DA) for anti-dementia drugs (rivastigmine, donepezil and galantamine) were allocated to “central nervous system stimulants” thereby placing these medications under the PEACE category ‘no consensus.’ However Colloca et al. may have allocated the same ATC codes to “acetylcholinesterase inhibitors,” placing them under the PEACE category ‘never appropriate,’ and may explain the difference in proportions of residents receiving never appropriate medications between studies. However, despite such differences between inclusion criteria and methods, findings from studies using the PEACE criteria have in some cases been surprisingly consistent with three studies reporting the most commonly prescribed ‘never’ appropriate medications as anticholinesterase inhibitors and lipid-lowering agents [17, 20, 22].

The authors of several studies in our review interpreted their results as indicating that people with advanced dementia undergo excessive pharmacological treatment [15, 16, 20, 22]. The reasons speculated included a lack of evidence-based guidance for clinicians [15, 20–22], a hesitancy among health professionals to take patients off medications where the impact has not been formally evaluated in advanced dementia [15, 19, 21], and the possibility that prescribers may not have recognised advanced dementia as a terminal illness needing to be treated with a palliative approach [18]. It may also be that discussions about reducing medications are sometimes avoided by health professionals because they require acknowledgement that the person with dementia is nearing the end of life [23]. This particular challenge has been identified in other palliative populations across a range of settings. Collier et al. (2013) have created a model to provide a systematic framework for hospice clinicians to have difficult conversations with patients, families and interdisciplinary clinical colleagues about the need to change prescribing when clinical decline occurs [23]. While broadly developed for individuals receiving end of life care, it may be applied to individuals with advanced dementia in order to facilitate discussion and improve care.

The widely held view that polypharmacy is undesirable in advanced dementia and end of life care is consistent with evidence that number of medications is related to adverse outcomes such as delirium, cognitive decline and loss of appetite [24]. However, when used in isolation (as by Blass et al. [2008] [15]), number of medications is too simplistic to be a useful index of the safety and quality of prescribing in advanced dementia. Both Blass et al. themselves and Tjia et al. found that the type but not number of medications changed over time as individuals with advanced dementia approached death, and a cross-sectional study has found that patients taking fewer than eight medications were more likely to be *underusing* a potentially useful medication [25]. The aim of palliative prescribing is to support comfort and quality of life, and in many cases, medications may need to be added to mitigate symptoms [23].

Reducing numbers of medications at the end of life also requires due attention to complexities inherent in deprescribing. While medications can be withdrawn safely, there is a risk of withdrawal reactions, symptom recurrence or reactivation of underlying disease [26]. Evidence is lacking in advanced dementia, however there is a growing body of research on the potential benefits of deprescribing in older people more generally [26]. It was shown that medication classes for secondary prevention such as lipid-lowering agents, antibiotics, antihypertensives and psychotropics can be withdrawn in older patients without causing harm. A system has been developed to inform deprescribing in disabled older adults in the form of an algorithm for decision-making [27]. Drug discontinuation based on this algorithm has been found not to increase significant adverse events, and only 10 % of the drugs ceased had to be readministered because of the return of the original indication for the drug. The same authors also tested their deprescribing algorithm in older adult community dwellers and were able to successfully deprescribe medications in 81 % of their sample with no significant adverse events or deaths attributable to discontinuation [28]. Future work is needed to examine the applicability of this algorithm to people with advanced dementia specifically and adapt as necessary.

First and foremost, this review is limited by the small pool of studies found that have focused on identifying potentially inappropriate medications in people with advanced dementia, limiting the avenues available for synthesis and conclusion. In particular, the absence of any studies validating systems against clinical outcomes necessarily limits the evidence base for improving the safety and quality of medication use in advanced dementia. Methodological limitations of the review include not requiring the primary aim of included studies to match those of this review and the possibility that we may not have identified all relevant research in the field. Whilst we expanded the search terms used by a previous review [9] and took a systematic approach to inclusion/exclusion, articles in the field of deprescribing are notoriously difficult to find [29], and nearly half the articles included in this study were found through hand searching rather than through databases.

## Conclusion

While there are well-accepted criteria available for identifying potentially inappropriate prescribing in older adults, these cannot be readily applied to the case of advanced dementia, where there are disease-specific concerns and a palliative approach is needed. The PEACE criteria show promise for further development, but require further studies to elucidate how decision-making should be informed by individual clinical context and how considerations may differ between deprescribing versus initiation. Further studies are also needed to identify potentially inappropriate medications with reference to empirical data on adverse events and other negative outcomes, rather than solely relying on the perceptions of health professionals and data and theory relating to standard pharmacological theory. Finally, studies are needed to test the ability of systems to identify potentially inappropriate prescribing to improve the quality and safety of medication use in people with advanced dementia.

## Abbreviations

AMED, Allied and Complementary Medicine Database; ATC, Anatomical Therapeutic Classification system; CINAHL, Cumulative Index to Nursing and Allied Health Literature; CPS, Cognitive Performance Scale; FAST, Functional Assessment Staging Tool (FAST); MeSH, Medical Subject Headings; PEACE, Palliative Excellence in Alzheimer Care Efforts program; PRISMA, Systematic Reviews and Meta-analyses statement; STROBE, STrengthening the Reporting of OBservational studies in Epidemiology checklist
